# Psychological resilience profiles and their psychosocial correlates among adults with prediabetes: a latent profile analysis

**DOI:** 10.3389/fpsyt.2026.1934725

**Published:** 2026-09-24

**Authors:** Xiling Hu, Hui Liu, Kunhua Yu, Xinqi Zhuang, Jianzhong Zhang, Yin-Ping Zhang

**Affiliations:** 1 School of Nursing, Xi’an Jiaotong University Health Science Center, Xi’an, China; 2 Department of Endocrinology and Metabolism, Guangdong Provincial Key Laboratory of Diabetology and Guangzhou Municipal Key Laboratory of Mechanistic and Translational Obesity Research, The Third Affiliated Hospital of Sun Yat-sen University, Guangzhou, China

**Keywords:** determinants, latent profile analysis, mental health, prediabetes, psychological resilience

## Abstract

**Background:**

Prediabetes is a high-risk metabolic state with a rapidly increasing global prevalence. Beyond its metabolic consequences, individuals with prediabetes face an elevated risk of depression and psychological distress. Psychological resilience, a key protective factor for mental health, may play an important role; however, its heterogeneity and associated factors in this population remain unclear. This study aimed to identify latent profiles of psychological resilience and examine their associated factors within this population.

**Methods:**

This cross-sectional study enrolled 315 adult patients with prediabetes. Data were collected using validated questionnaires designed to evaluate psychological resilience (CD-RISC-10), self-efficacy (GSES), social support (SSRS), and illness perception (BIPQ). Latent profile analysis (LPA) was performed in Mplus 8.3 to identify unobserved subgroups of psychological resilience. Model fit was evaluated using AIC, BIC, adjusted BIC, entropy,the BLRT, and the LMR-LRT, together with interpretability. Multinomial logistic regression analysis was conducted to identify factors associated with latent profile membership.

**Results:**

The mean psychological resilience score among adults with prediabetes was 28.56 ± 7.19, indicating a moderate level. Latent profile analysis identified three distinct subgroups of psychological resilience: the low-resilience profile (n = 58, 18.4%), the moderate-resilience profile (n = 152, 48.3%), and the high-resilience profile (n = 105, 33.3%), indicating marked heterogeneity in resilience patterns. Multinomial logistic regression analysis showed that self-efficacy, social support, medical payment method, and marital status were independently associated with latent profile membership.

**Conclusion:**

This study identified three distinct latent profiles of psychological resilience among adults with prediabetes, revealing substantial heterogeneity within this population. Self-efficacy, social support, marital status, and medical payment method are key factors associated with profile membership. These findings highlight the importance of incorporating psychological and social determinants into targeted nursing interventions to enhance resilience in this population.

## 1 Introduction

Prediabetes is an intermediate metabolic state between normoglycemia and diabetes, encompassing impaired fasting glucose (IFG), impaired glucose tolerance (IGT), or their combination (IFG + IGT) (1). It is characterized by elevated blood glucose levels that do not yet meet the diagnostic criteria for diabetes. With rapid population aging and lifestyle transitions, its prevalence has risen markedly, establishing prediabetes as a major global public health concern. According to the International Diabetes Federation (2), approximately 21.2% of adults worldwide were affected in 2024, with projections rising to 22.7% by 2050. In China, the prevalence is even higher, reaching 35.2% among adults (3). In addition to its high risk of progression to diabetes, prediabetes is associated with a broad range of adverse outcomes, including cardiovascular disease, microvascular complications, malignancies, and cognitive decline (1).

Beyond its well-established metabolic and clinical consequences, accumulating evidence indicates that prediabetes is also associated with a substantial psychological burden (4). A meta-analysis reported a modestly increased risk of depression in individuals with prediabetes (*OR* = 1.11), with a dose-response pattern showing progressively higher risk as glucose metabolism deteriorates, particularly among younger populations (5). This association has been consistently observed across diverse settings. For instance, a study conducted in China reported a significantly elevated risk of depression among individuals with prediabetes (*OR* = 1.53), while data from the Korean National Health and Nutrition Examination Survey similarly demonstrated a higher prevalence of depressive symptoms compared with normoglycemic individuals (6, 7). In addition to depression, individuals with prediabetes have been reported to exhibit higher levels of anxiety than both normoglycemic individuals and those with diagnosed diabetes (8, 9). Collectively, these findings suggest increased vulnerability to psychological distress in this population, underscoring the substantial psychological burden associated with prediabetes. Moreover, emerging evidence indicates a bidirectional relationship between psychological and metabolic processes, whereby psychological distress-including chronic depression, anxiety, and stress-may contribute to metabolic deterioration. Mechanistically, stress-related activation of the sympathetic nervous system and the hypothalamic-pituitary-adrenal (HPA) axis can increase cortisol secretion, thereby promoting insulin resistance and elevating the risk of type 2 diabetes (10).

Despite growing recognition of these bidirectional interactions, psychological adaptation in individuals with prediabetes remains insufficiently understood. Psychological resilience is generally defined as the capacity to maintain or regain psychological well-being when confronted with adversity, stress, or significant life challenges (11). Although resilience was initially regarded as a relatively stable personality trait, it is now widely conceptualized as a dynamic and modifiable process of positive adaptation that arises from the interaction between individual characteristics and environmental resources (12). This process encompasses multiple psychological capacities, including perseverance, optimism, adaptive coping, emotional regulation, and cognitive flexibility, which enable individuals to effectively respond to adversity and maintain psychological functioning (13). As an important psychological resource, resilience has been identified as a key protective factor for promoting health and well-being (11). Higher levels of resilience have been associated with reduced risks of diabetes incidence and all-cause mortality, as well as improved physical functioning and quality of life (14, 15). In chronic disease populations, resilience mitigates psychological distress and promotes mental health through enhanced coping and emotional regulation (13). However, resilience is inherently heterogeneous, with substantial inter-individual variability suggesting that individuals may exhibit distinct adaptive patterns in response to similar stressors. This underscores the need to move beyond variable-centered approaches and examine the heterogeneity of resilience within the prediabetes population.

Latent profile analysis (LPA) is a person-centered statistical approach that identifies unobserved subgroups within a population based on individual response patterns (16). Unlike variable-centered methods, LPA classifies individuals into distinct profiles, thereby capturing heterogeneity in psychological characteristics that may be obscured in conventional analyses (17). Given the variability in psychological resilience among individuals with prediabetes, LPA offers a robust framework for delineating distinct resilience patterns and uncovering underlying heterogeneity. However, empirical evidence on resilience profiles and their associated factors in this population remains limited. From a theoretical perspective, the Health Ecological Model proposes that health is jointly determined by individual characteristics and environmental factors across multiple ecological levels, emphasizing that health outcomes result from interactions between individuals and their surrounding environment (18). This model has been widely applied to identify multilevel determinants of health and disease. It comprises five hierarchical levels: innate personal traits, behavioral characteristics, interpersonal networks, living and working conditions, and policy environment. Guided by this framework, the present study selected variables from each ecological level to comprehensively examine factors associated with psychological resilience among adults with prediabetes. Specifically, the innate personal traits level included demographic and health-related characteristics (e.g., age, sex, body mass index, family history of diabetes, and comorbidities); the behavioral characteristics level included smoking and alcohol consumption; the interpersonal network level included marital status, living arrangements, place of residence, and perceived social support; the living and working conditions level included educational attainment, occupation, household income, and religious affiliation; and the policy environment level was represented by health insurance type. In addition, self-efficacy and illness perception were incorporated as important psychological factors because substantial evidence has demonstrated their important roles in psychological resilience and adaptation to chronic disease. Therefore, this study aimed to identify latent profiles of psychological resilience among adults with prediabetes using latent profile analysis and to examine the multilevel factors associated with profile membership based on the Health Ecological Model.

## 2 Materials and methods

### 2.1 Research design

This study utilized a cross-sectional survey design and implemented a convenience sampling strategy to recruit individuals with prediabetes who attended The Third Affiliated Hospital of Sun Yat-sen University in Guangzhou from March 2024 to February 2025. Furthermore, the research adhered to the Strengthening the Reporting of Observational Studies in Epidemiology (STROBE) guidelines for cross-sectional studies (see **Supplementary Table 1**) (19).

### 2.2 Participants

The inclusion criteria were as follows (1): fulfilment of the diagnostic criteria for prediabetes as outlined in the Chinese Expert Consensus on Intervention for Prediabetes in Adults (1) (2); age of 18 years or older (3); sufficient communication and comprehension abilities; and (4) provision of informed consent with voluntary participation. The exclusion criteria were (1): critically ill individuals or those with severe comorbid conditions that would prevent survey completion (2); individuals with cognitive impairments or psychiatric disorders that hindered accurate understanding or completion of the questionnaire; and (3) cases with missing or incomplete data. This study received approval from the Ethics Committee of The Third Affiliated Hospital of Sun Yat-sen University (Approval No.: RG2023-262-02).

### 2.3 Sample size

The sample size was determined in accordance with established guidelines for latent profile analysis (LPA) and regression modeling. Previous research indicates that a minimum sample size of 200–300 is necessary for stable estimations in LPA, with larger samples yielding more reliable class solutions (20, 21). Furthermore, the sample size was deemed sufficient for multinomial logistic regression, adhering to the principle of at least 10 observations per predictor variable (22). Consequently, a minimum sample size of 300 participants was mandated for this study.

### 2.4 Measures

#### 2.4.1 Sociodemographic and disease-related factors

A self-administered, purpose-designed questionnaire was utilized to systematically gather sociodemographic characteristics and disease-related information. The sociodemographic variables included age, sex, marital status, educational attainment, occupation, per capita monthly household income, type of health insurance, place of residence, living arrangements, and religious affiliation. Disease-related variables comprised body mass index (BMI), duration of prediabetes, family history, and comorbid conditions such as hypertension, coronary heart disease, hyperuricemia, and hyperlipidemia. Additionally, lifestyle factors were assessed, including smoking and alcohol consumption. Height and weight were measured using a standardized stadiometer and calibrated scale, with BMI calculated as weight (kg) divided by height squared (m²).

#### 2.4.2 The 10-item connor-davidson resilience scale

The 10-item Connor-Davidson Resilience Scale (CD-RISC-10) was used to assess patients’ psychological resilience. The original 25-item Connor-Davidson Resilience Scale was developed by Connor and Davidson in 2003 and comprises five dimensions (13). However, subsequent psychometric evaluation by Campbell-Sills and Stein (23) indicated limited support for the original five-factor structure, leading to the development of a 10-item unidimensional version with favorable psychometric properties. The Chinese version was translated and psychometrically validated by Zhang et al. (24). Each item is rated on a 5-point Likert scale ranging from 0 (“not at all”) to 4 (“true nearly all the time”), yielding a total score of 0 - 40, with higher scores indicating greater psychological resilience. Previous studies have demonstrated acceptable reliability of the CD-RISC-10, including a Cronbach’s α of 0.85 (23). Although population-specific psychometric validation of the CD-RISC-10 among adults with prediabetes remains limited, the instrument has been widely used to assess psychological resilience across diverse adult and clinical populations, and its conceptual relevance to psychological adaptation in individuals with prediabetes supports its use in the present study. In the present study, the CD-RISC-10 demonstrated excellent internal consistency, with a Cronbach’s α of 0.930.

#### 2.4.3 The general self-efficacy scale

The General Self-Efficacy Scale (GSES) was used to assess patients’ self-efficacy. The scale was originally developed by Schwarzer and Jerusalem (25) and later translated and validated in Chinese by Wang et al. (26). It measures individuals’ perceived confidence in their ability to cope with environmental demands and manage a variety of stressful situations. The GSES consists of 10 items rated on a 4-point Likert scale ranging from 1 (“not at all true”) to 4 (“exactly true”), with total scores ranging from 10 to 40; higher scores indicate stronger self-efficacy. Previous studies have demonstrated satisfactory psychometric properties, including a Cronbach’s α of 0.87 and a test-retest reliability of 0.83 (26). In the present study, the scale demonstrated excellent internal consistency, with a Cronbach’s alpha of 0.911.

#### 2.4.4 The social support rating scale

The Social Support Rating Scale (SSRS) was adopted in the present study to quantify patients’ perceived level of social support. This instrument, developed by Xiao (27), comprises 10 items organized into three domains: subjective support, objective support, and utilization of social support. Items 1–4 and 8–10 are assessed using a 4-point Likert response format. Item 5 is a multi-response item that captures perceived support from five sources (spouse, children, parents, siblings, and others), with each source rated from 1 (“none”) to 4 (“full support”). Items 6–7 are scored as 0 when no source is reported, whereas each identified source contributes 1 point to the total score. Overall scores range from 12 to 66, with higher values reflecting stronger social support. The SSRS has been widely validated in Chinese populations and demonstrates robust psychometric performance, with Cronbach’s α coefficients for the total scale and its sub-dimensions between 0.89 and 0.94, alongside a test-retest reliability of 0.92 (27).

#### 2.4.5 The brief iIllness perception questionnaire

The Brief Illness Perception Questionnaire (BIPQ), originally developed by Broadbent et al. (28), was used in the present study in its Chinese version translated and culturally adapted by Sun et al. (29). The instrument consists of nine items. Items 1–5 assess cognitive representations of illness, including perceived consequences on daily life, timeline, personal control, treatment control, and symptom identity. Items 6–8 evaluate emotional and cognitive appraisals, encompassing concern about the illness, perceived understanding, and emotional impact. Items 1–8 are rated on an 11-point scale ranging from 0 to 10, with Items 3, 4, and 7 reverse-scored to ensure consistent directionality. The total score ranges from 0 to 80, with higher scores indicating more negative illness perceptions. Item 9 is an open-ended question used to elicit causal attributions and is excluded from scoring. The Chinese version has demonstrated satisfactory psychometric properties, with a Cronbach’s α of 0.831 and a test-retest reliability of 0.931 (29).

### 2.5 Data collection

Following approval from the hospital ethics committee, eligible participants were identified through electronic medical record screening based on predefined inclusion and exclusion criteria. The research team provided a comprehensive explanation of the study objectives, significance, and procedures, and responded to participants’ inquiries to alleviate potential psychological concerns. Written informed consent was obtained from all participants prior to data collection in full respect of the principle of autonomy. Questionnaires were administered face-to-face by a trained nursing graduate student in a quiet, private environment, with standardized instructions to ensure independent completion, and participants were informed that there were no right or wrong answers and that their responses would be kept confidential and used solely for research purposes. The investigator clarified the meaning of questionnaire items only when necessary and avoided suggesting or influencing participants’ responses. For participants unable to complete the questionnaire independently, structured interviews were conducted using the same standardized instructions, and responses were recorded verbatim by the investigator. Each completed questionnaire was reviewed immediately for completeness, and respondents were prompted to supply any missing information when necessary. In addition, routine quality control and data verification procedures were implemented to ensure accuracy and integrity of the dataset. A total of 320 questionnaires were distributed, of which 315 were valid, yielding an effective response rate of 98.44%.

### 2.6 Data analysis

Statistical analyses were performed using SPSS version 27.0 (IBM Corp., Armonk, NY, USA). Continuous variables following a normal distribution are presented as mean ± standard deviation (*SD*), whereas non-normally distributed variables are expressed as median (interquartile range, *IQR*). Categorical variables are summarized as frequencies and percentages (*%*). Latent profile analysis (LPA) was conducted using the tidyLPA package in R (version 4.4.2). The 10 items of the Connor-Davidson Resilience Scale (CD-RISC-10) were entered as continuous observed indicators after z-standardization. Profile-specific means were freely estimated, whereas indicator variances were constrained to be equal across profiles and covariances among indicators were fixed to zero (Model 1 in tidyLPA), consistent with the assumption of local independence. Models with one to six latent profiles were estimated, and the optimal solution was determined by jointly considering statistical fit indices and substantive interpretability. Model fit was evaluated using the Akaike information criterion (AIC), Bayesian information criterion (BIC), and sample-size adjusted BIC (aBIC), with lower values indicating better model fit. The bootstrapped likelihood ratio test (BLRT) and the Lo-Mendell-Rubin adjusted likelihood ratio test (LMR-LRT) were employed to compare models, with *P* < 0.05 indicating that a k-class model fit significantly better than the k-1 class model. Entropy was used to assess classification precision, with values ≥ 0.80 indicating high classification accuracy. Univariate analyses were performed to describe and preliminarily compare the characteristics of participants across the latent profiles. Variables showing statistically significant differences in the univariate analyses (*P* < 0.05) were considered candidate variables and were subsequently entered into the multinomial logistic regression model. Continuous variables were compared using one-way analysis of variance (ANOVA), while categorical variables were examined using chi-square tests or Fisher’s exact tests, as appropriate. The multinomial regression analysis was conducted to identify factors independently associated with latent profile membership. Statistical significance was set at *P* < 0.05.

## 3 Results

### 3.1 General characteristics of participants

A total of 315 adults with prediabetes were enrolled in this study. The baseline sociodemographic and disease-related characteristics of the study participants are presented in **Table 1**.

**Table 1:** Baseline characteristics of adults with prediabetes (*N* = 315).

Variable	Total (n=315)	Variable	Total (n=315)
Age (years), n (%)		BMI (kg/m²), n (%)	
<50	140 (44.44)	<24	106 (33.65)
≥50	175 (55.56)	≥24	209 (66.35)
Sex, n (%)		Clinical diagnosis, n (%)	
Male	151 (47.94)	IFG	33 (10.48)
Female	164 (52.06)	IGT	249 (79.04)
Marital status, n (%)		IFG+IGT	33 (10.48)
Single	70 (22.22)	Disease duration (months), n (%)	
Married	230 (73.02)	<6	149 (47.3)
Divorced/Widowed	15 (4.76)	≥6	166 (52.7)
Education level, n (%)		Family history of diabetes, n (%)	
Primary school or below	48 (15.24)	No	220 (69.84)
Secondary school	113 (35.87)	Yes	95 (30.16)
College	43 (13.65)	Hypertension, n (%)	
Bachelor’s degree	94 (29.84)	No	220 (69.84)
Master’s degree or above	17 (5.4)	Yes	95 (30.16)
Occupation, n (%)		Coronary heart disease, n (%)	
Non-manual labor	123 (39.05)	No	270 (85.71)
Manual labor	192 (60.95)	Yes	45 (14.29)
Monthly household income per capita (RMB), n (%)		Hyperuricemia, n (%)	
<5000	94 (29.84)	No	278 (88.25)
5000~10000	132 (41.91)	Yes	37 (11.75)
10000~15000	43 (13.65)	Hyperlipidemia, n (%)	
≥15000	46 (14.6)	No	263 (83.49)
Medical payment method, n (%)		Yes	52 (16.51)
Self-paid	31 (9.84)	Smoking status, n (%)	
Public-funded	21 (6.67)	No	258 (81.9)
Medical insurance	263 (83.49)	Yes	57 (18.1)
Residence, n (%)		Alcohol consumption, n (%)	
Urban	271 (86.03)	No	277 (87.94)
Rural	44 (13.97)	Yes	38 (12.06)
Living arrangement, n (%)		Psychological resilience (Mean ± *SD*)	28.562 ± 7.186
Living alone	42 (13.33)	Self-efficacy (Mean ± *SD*)	27.590 ± 6.359
Living with others	273 (86.67)	Social support (Mean ± *SD*)	38.686 ± 7.177
Religious belief, n (%)		Illness perception (Mean ± *SD*)	40.092 ± 9.452
No	305 (96.83)		
Yes	10 (3.17)		

Data are presented as *n* (%) or mean (Standard Deviation, SD).

BMI, Body Mass Index; IFG, impaired fasting glucose; IGT, impaired glucose tolerance.

### 3.2 Latent profile model selection

Latent profile models comprising one to six profiles were estimated, and the corresponding model fit indices are presented in **Table 2**. As the number of profiles increased, the Akaike Information Criterion (AIC) and simple size-adjusted Bayesian Information Criterion (aBIC) decreased monotonically. The three-profile model was selected as the optimal solution because it demonstrated the highest entropy (0.919), relatively lower AIC, BIC, and adjusted BIC (aBIC) values, and statistically significant bootstrap likelihood ratio test (BLRT) and Lo-Mendell-Rubin adjusted likelihood ratio test (LMR-LRT) results (*P* < 0.01). In addition to these statistical fit indices, model selection also considered profile size, interpretability, and clinical relevance. The three identified profiles each contained an adequate proportion of participants and exhibited distinct and readily interpretable patterns of psychological resilience, supporting meaningful stratification of adults with prediabetes according to resilience level. This classification may facilitate the identification of individuals with different psychological support needs and inform the development of tailored intervention strategies. Therefore, considering both statistical performance and clinical interpretability, the three-profile solution was retained for subsequent analyses.

**Table 2:** Fit indices of latent profile models for psychological resilience among adults with prediabetes (*N* = 315).

Model	Log-likelihood	AIC	BIC	aBIC	BLRT (*p*)	LMR-LRT (*p*)	Entropy	Class probabilities
1-profile	-4464.648	8969.297	9044.348	8980.914	–		1	–
2-profile	-3869.938	7801.875	7918.205	7819.881	< 0.010	< 0.001	0.898	0.362/0.638
3-profile	-3620.222	7324.444	7482.052	7348.839	< 0.010	< 0.001	0.919	0.184/0.483/0.333
4-profile	-3567.410	7240.821	7439.707	7271.606	< 0.010	< 0.001	0.848	0.171/0.372/0.289/0.168
5-profile	-3503.935	7135.870	7376.035	7173.044	< 0.010	< 0.001	0.857	0.063/0.333/0.308/0.140/0.156
6-profile	-3484.845	7119.689	7401.132	7163.253	< 0.010	< 0.001	0.859	0.063/0.318/0.044/0.149/0.140/0.286

AIC, Akaike information criterion; BIC, Bayesian information criterion; aBIC, sample size-adjusted BIC; BLRT, bootstrap likelihood ratio test; LMR-LRT, Lo-Mendell-Rubin adjusted likelihood ratio test.

### 3.3 Characteristics of the identified psychological resilience profiles

Based on the overall response patterns across the 10 CD-RISC-10 items, the three latent profiles were interpreted as representing low, moderate, and high levels of psychological resilience and were accordingly labeled as the low-resilience profile, moderate-resilience profile, and high-resilience profile, respectively (**Figure 1**). The corresponding mean CD-RISC-10 total scores were 17.19 ± 3.73, 27.68 ± 2.59, and 36.12 ± 2.75 for the low-, moderate-, and high-resilience profiles, respectively (**Table 3**). The low-resilience profile (P1) comprised 58 participants (18.4%) and was characterized by consistently low scores across all CD-RISC-10 items. The moderate-resilience profile (P2) included 152 participants (48.3%) and exhibited intermediate scores across all items. The high-resilience profile (P3) consisted of 105 participants (33.3%) and showed consistently high scores across all items.

**Figure 1:**
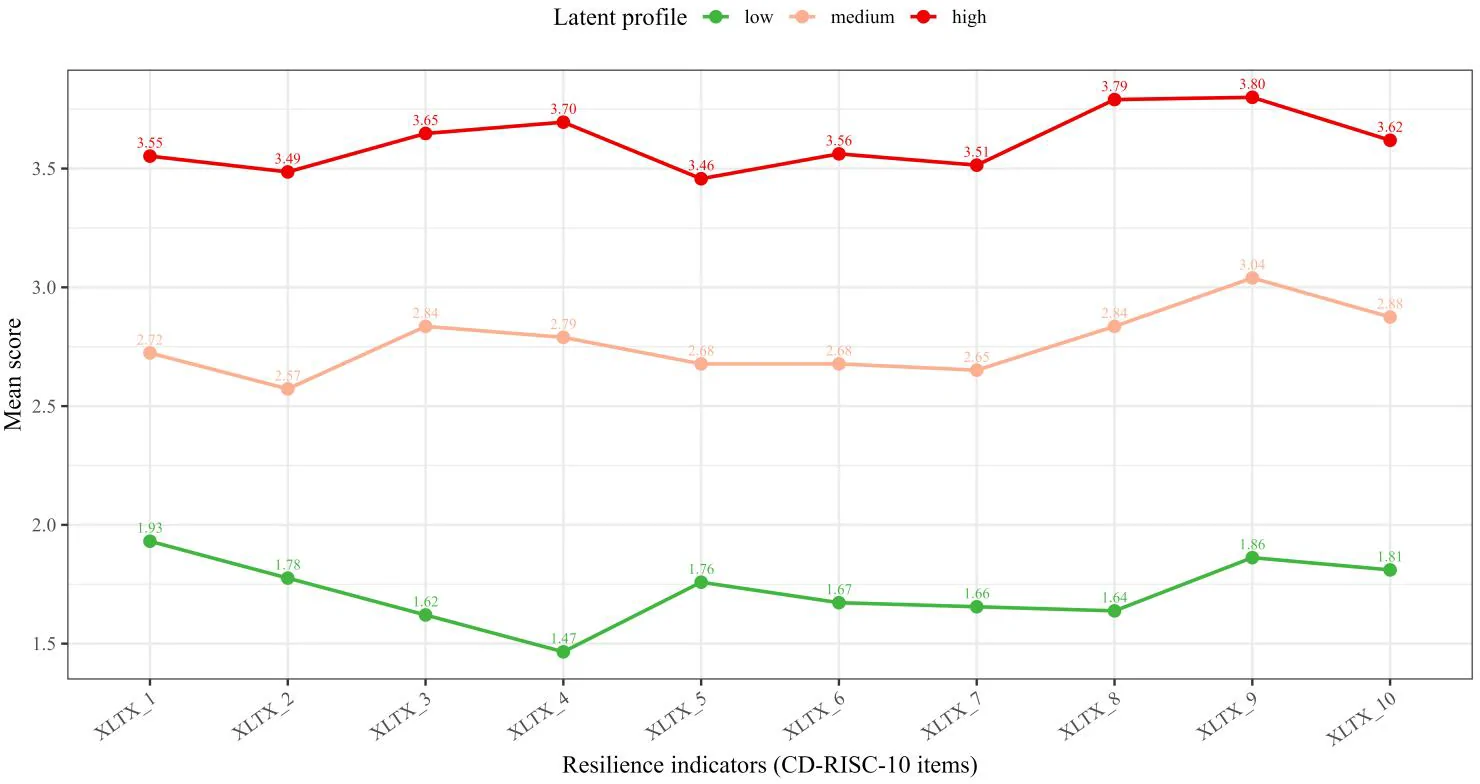
Mean CD-RISC-10 item scores across the three identified psychological resilience profiles. Values represent mean scores for individual items within each profile.

**Table 3:** Comparison of participant characteristics across the identified psychological resilience profiles among adults with prediabetes (*N* = 315).

Variable	P1 (n=58)	P2 (n=152)	P3 (n=105)	Test statistic	*P*
Age (years), n (%)				11.436	0.003
<50	34 (58.62)	72 (47.37)	34 (32.38)		
≥50	24 (41.38)	80 (52.63)	71 (67.62)		
Sex, n (%)				5.937	0.051
Male	20 (34.48)	74 (48.68)	57 (54.29)		
Female	38 (65.52)	78 (51.32)	48 (45.71)		
Marital status, n (%)				36.974	<0.001
Single	26 (44.83)	35 (23.03)	9 (8.57)		
Married	27 (46.55)	115 (75.66)	88 (83.81)		
Divorced/Widowed	5 (8.62)	2 (1.31)	8 (7.62)		
Education level, n (%)				5.815	0.668
Primary school or below	11 (18.97)	22 (14.47)	15 (14.29)		
Secondary school	18 (31.03)	56 (36.85)	39 (37.14)		
College	6 (10.34)	23 (15.13)	14 (13.33)		
Bachelor’s degree	22 (37.94)	43 (28.29)	29 (27.62)		
Master’s degree or above	1 (1.72)	8 (5.26)	8 (7.62)		
Occupation, n (%)				1.661	0.436
Non-manual labor	20 (34.48)	57 (37.5)	46 (43.81)		
Manual labor	38 (65.52)	95 (62.5)	59 (56.19)		
Monthly household income per capita (RMB), n (%)				7.315	0.293
<5000	19 (32.76)	46 (30.26)	29 (27.62)		
5000~10000	24 (41.38)	69 (45.40)	39 (37.15)		
10000~15000	9 (15.52)	20 (13.16)	14 (13.33)		
≥15000	6 (10.34)	17 (11.18)	23 (21.9)		
Medical payment method, n (%)				16.239	0.003
Self-paid	13 (22.41)	13 (8.55)	5 (4.76)		
Public-funded	1 (1.72)	10 (6.58)	10 (9.52)		
Medical insurance	44 (75.87)	129 (84.87)	90 (85.72)		
Residence, n (%)				5.460	0.065
Urban	49 (84.48)	125 (82.24)	97 (92.38)		
Rural	9 (15.52)	27 (17.76)	8 (7.62)		
Living arrangement, n (%)				7.180	0.028
Living alone	12 (20.69)	23 (15.13)	7 (6.67)		
Living with others	46 (79.31)	129 (84.87)	98 (93.33)		
Religious belief, n (%)				1.403	0.496
No	57 (98.28)	148 (97.37)	100 (95.24)		
Yes	1 (1.72)	4 (2.63)	5 (4.76)		
BMI (kg/m²), n (%)				2.274	0.321
<24	15 (25.86)	56 (36.84)	35 (33.33)		
≥24	43 (74.14)	96 (63.16)	70 (66.67)		
Clinical diagnosis, n (%)				6.673	0.154
IFG	4 (6.9)	15 (9.87)	14 (13.33)		
IGT	50 (86.2)	115 (75.66)	84 (80)		
IFG+IGT	4 (6.9)	22 (14.47)	7 (6.67)		
Disease duration (months), n (%)				2.700	0.259
<6	26 (44.83)	79 (51.97)	44 (41.9)		
≥6	32 (55.17)	73 (48.03)	61 (58.1)		
Family history of diabetes, n (%)				0.659	0.719
No	43 (74.14)	104 (68.42)	73 (69.52)		
Yes	15 (25.86)	48 (31.58)	32 (30.48)		
Hypertension, n (%)				1.033	0.597
No	43 (74.14)	107 (70.39)	70 (66.67)		
Yes	15 (25.86)	45 (29.61)	35 (33.33)		
Coronary heart disease, n (%)				10.080	0.006
No	54 (93.1)	135 (88.82)	81 (77.14)		
Yes	4 (6.9)	17 (11.18)	24 (22.86)		
Hyperuricemia, n (%)				0.203	0.903
No	52 (89.66)	133 (87.5)	93 (88.57)		
Yes	6 (10.34)	19 (12.5)	12 (11.43)		
Hyperlipidemia, n (%)				1.196	0.550
No	47 (81.03)	125 (82.24)	91 (86.67)		
Yes	11 (18.97)	27 (17.76)	14 (13.33)		
Smoking status, n (%)				0.888	0.642
No	50 (86.21)	123 (80.92)	85 (80.95)		
Yes	8 (13.79)	29 (19.08)	20 (19.05)		
Alcohol consumption, n (%)				1.318	0.517
No	49 (84.48)	133 (87.5)	95 (90.48)		
Yes	9 (15.52)	19 (12.5)	10 (9.52)		
Psychological resilience (Mean ± *SD*)	17.190 ± 3.730	27.678 ± 2.588	36.124 ± 2.748	820.754	<0.001
Self-efficacy (Mean ± *SD*)	21.862 ± 5.564	26.961 ± 4.807	31.667 ± 5.993	64.347	<0.001
Social support (Mean ± *SD*)	33.793 ± 7.145	38.454 ± 6.802	41.724 ± 6.134	26.727	<0.001
Illness perception (Mean ± *SD*)	42.517 ± 10.330	40.414 ± 8.649	38.286 ± 9.795	3.989	0.019

P1, the low-resilience profile; P2, the moderate-resilience profile; P3, the high-resilience profile. Data are presented as *n* (%) or mean (Standard Deviation, SD).

BMI, Body Mass Index; IFG, impaired fasting glucose; IGT, impaired glucose tolerance.

### 3.4 Univariate analysis of latent profiles of psychological resilience in adults with prediabetes

The psychological resilience scores ranged from 8 to 40, with a mean of 28.56 ± 7.19. Univariate analyses revealed significant differences among the three latent profiles with respect to age, marital status, medical payment method, living arrangement, presence of coronary heart disease, self-efficacy, social support, and illness perception (all *P* < 0.05). Detailed results are presented in **Table 3**.

### 3.5 Multinomial logistic regression analysis of latent profiles in adults with prediabetes

Multinomial logistic regression analysis was performed with latent resilience profiles as the dependent variable, using the high-resilience profile (P3) as the reference category. Variables showing statistically significant associations with latent profile membership in the univariate analyses were subsequently entered simultaneously into the multinomial logistic regression model using the enter method. The results are presented in **Table 4**. For the low-resilience profile (P1), medical payment method, self-efficacy, and social support were identified as significant independent factors. Compared with self-paying participants, those covered by public medical insurance (*OR* = 0.01, 95% *CI*: 0.00-0.30, *P* = 0.008) or medical insurance schemes (*OR* = 0.22, 95% *CI*: 0.05-0.92, *P* = 0.038) were less likely to be classified into the low-resilience profile. In addition, higher self-efficacy (*OR* = 0.69, 95% *CI*: 0.63-0.76, *P* < 0.001) and higher social support (*OR* = 0.88, 95% *CI*: 0.82-0.96, *P* = 0.002) were associated with a less likelihood of belonging to the low-resilience profile. For the moderate-resilience profile (P2), self-efficacy (*OR* = 0.87, 95% *CI*: 0.82-0.92, *P* < 0.001) and social support (*OR* = 0.95, 95% *CI*: 0.90-1.00, *P* = 0.037) remained significantly associated with profile membership. Additionally, divorced or widowed participants were less likely to be classified into this profile compared with single participants (*OR* = 0.13, 95% *CI*: 0.02-0.89, *P* = 0.037). No significant associations were observed between age, living arrangement, coronary heart disease, or illness perception and latent profile membership (all *P* > 0.05).

**Table 4:** Multinomial logistic regression analysis of latent profiles of psychological resilience among adults with prediabetes (*N* = 315).

Variable	Low-resilience profile (P1) vs high-resilience profile (P3)						Moderate-resilience profile (P2) vs high-resilience profile (P3)					
	*β*	*SE*	Wald *χ^2^*	*OR*	95% *CI*	*p*-value	*β*	*SE*	Wald *χ^2^*	*OR*	95% *CI*	*p*-value
Intercept	14.17	2.29	6.19	1429561.19	(16048.63, 127340789.16)	<0.001	6.86	1.57	4.37	950.55	(43.93, 20569.47)	<0.001
Age (years)												
<50	reference											
≥50	0.21	0.63	0.34	1.24	(0.36, 4.26)	0.737	-0.27	0.37	-0.73	0.76	(0.37, 1.57)	0.464
Sex												
Male	reference											
Female	0.74	0.48	1.56	2.10	(0.83, 5.32)	0.119	0.16	0.30	0.54	1.18	(0.65, 2.14)	0.590
Marital status												
Single	reference											
Married	-1.36	0.80	-1.70	0.26	(0.05, 1.23)	0.088	-0.23	0.57	-0.40	0.80	(0.26, 2.43)	0.688
Divorced/Widowed	-0.85	1.09	-0.78	0.43	(0.05, 3.63)	0.436	-2.07	1.00	-2.08	0.13	(0.02, 0.89)	0.037
Medical payment method												
Self-paid	reference											
Public-funded	-4.56	1.71	-2.67	0.01	(0.00, 0.30)	0.008	-0.75	0.80	-0.94	0.47	(0.10, 2.25)	0.346
Medical insurance	-1.50	0.72	-2.08	0.22	(0.05, 0.92)	0.038	-0.38	0.61	-0.62	0.68	(0.21, 2.27)	0.534
Residence												
Urban	reference											
Rural	1.08	0.68	1.60	2.96	(0.78, 11.18)	0.110	0.99	0.48	2.05	2.69	(1.05, 6.90)	0.040
Living arrangement												
Living alone	reference											
Living with others	-0.11	0.76	-0.15	0.89	(0.20, 3.96)	0.881	-0.44	0.61	-0.71	0.65	(0.19, 2.15)	0.478
Coronary heart disease												
No	reference											
Yes	0.23	0.72	0.32	1.26	(0.31, 5.17)	0.750	-0.34	0.41	-0.81	0.71	(0.32, 1.60)	0.415
Self-efficacy	-0.37	0.05	-7.34	0.69	(0.63, 0.76)	<0.001	-0.14	0.03	-5.12	0.87	(0.82, 0.92)	<0.001
Social support	-0.12	0.04	-3.08	0.88	(0.82, 0.96)	0.002	-0.05	0.03	-2.08	0.95	(0.90, 1.00)	0.037
Illness perception	0.03	0.02	1.41	1.04	(0.99, 1.09)	0.157	0.02	0.02	1.37	1.02	(0.99, 1.06)	0.172

The high-resilience profile (P3) was used as the reference category. Values are presented as regression coefficient (*β*), standard error (*SE*), odds ratio (*OR*), and 95% confidence interval (*CI*). *OR* represent the likelihood of membership in the low-resilience profile (P1) or the moderate-resilience profile (P2) relative to the high-resilience profile (P3).

## 4 Discussion

This study found that psychological resilience among adults with prediabetes was at a moderate level (28.56 ± 7.19), slightly higher than that reported in certain cohorts of patients with type 2 diabetes mellitus (26.61 ± 2.56) (30). This difference may be associated with the earlier stage of disease progression and relatively lower symptom burden in individuals with prediabetes, which may be linked to greater availability of adaptive psychological resources. However, the observed level was lower than that reported in other populations with chronic diseases (30.07  ±  7.12), suggesting potential room for improvement in psychological resilience among individuals with prediabetes and diabetes (31). This discrepancy may be related to differences in disease perception, coping experience, and illness duration across populations. Furthermore, LPA identified three distinct subgroups: the low-resilience profile (n = 58, 18.4%), the moderate-resilience profile (n = 152, 48.3%), and the high-resilience profile (n = 105, 33.3%), highlighting substantial heterogeneity in resilience patterns within this population. In addition, self-efficacy, social support, medical payment method, and marital status were significantly associated with latent profile membership. These findings suggest that these factors are associated with differences in psychological resilience profiles and may be useful for identifying subgroups that could benefit from tailored nursing assessment and intervention.

In this study, 33.3% of adults with prediabetes were classified into the high-resilience profile, whereas 18.4% were assigned to the low-resilience profile. Notably, nearly half of the participants (48.3%) were classified into the moderate-resilience profile. This distribution suggests substantial heterogeneity in psychological resilience within this population, with most individuals demonstrating an intermediate level of resilience. Such heterogeneity may be associated with differences in psychological adaptation during the early stage of chronic disease. Although the three identified profiles primarily differed in their overall resilience levels, they were derived using a person-centered latent profile analysis based on response patterns across all ten CD-RISC-10 items rather than arbitrary categorization according to total resilience scores. This approach provides a data-driven classification of individuals with similar response patterns and complements traditional variable-centered analyses by identifying latent subgroups with potentially different psychological support needs. Similar heterogeneity has been reported in other populations with chronic diseases, although differences in profile distributions across studies may be associated with variations in disease stage, age composition, measurement instruments, and study populations (31). From a clinical perspective, resilience profiling may provide useful information for risk stratification and individualized psychological assessment among adults with prediabetes. Specifically, individuals in the low-resilience profile may warrant more comprehensive psychological assessment and closer follow-up because they may be more likely to experience psychosocial difficulties when adapting to prediabetes. Those in the moderate-resilience profile, representing nearly half of the study population, may benefit from regular monitoring and supportive strategies aimed at strengthening psychological resources and maintaining adaptive coping. In contrast, individuals in the high-resilience profile may require continued reinforcement of existing positive coping resources and support for sustaining long-term healthy self-management behaviors. Therefore, incorporating resilience profiles into routine clinical assessment may assist nurses in developing more individualized and profile-informed health management strategies. However, because this study employed a cross-sectional design, longitudinal and intervention studies are warranted to determine whether resilience profile-guided management can improve psychological and health-related outcomes.

Using the high-resilience profile as the reference, lower self-efficacy was associated with a greater likelihood of belonging to the low-resilience profile or moderate-resilience profile, whereas higher self-efficacy was associated with membership in the high-resilience profile. This finding is consistent with previous studies reporting a positive association between self-efficacy and psychological resilience in individuals with chronic diseases (32, 33). According to Social Cognitive Theory, self-efficacy is regarded as an important determinant of health behaviors and psychological adaptation, providing a plausible theoretical framework for understanding the observed association (34). Individuals with higher self-efficacy may perceive greater confidence in managing health-related challenges and engaging in self-management behaviors, generating reinforcing “mastery experiences” that establish a virtuous cycle between self-efficacy and psychological resilience. The present findings suggest that self-efficacy may represent an important psychosocial characteristic for distinguishing resilience profiles among adults with prediabetes. Therefore, incorporating self-efficacy assessment into routine psychological evaluation may help identify individuals who could require additional support. Interventions based on Social Cognitive Theory, such as goal setting, peer support, motivational interviewing, and self-monitoring, may be considered as potential approaches for supporting self-management and psychological adaptation.

Similarly, higher levels of social support were also associated with a greater likelihood of belonging to the high-resilience profile, which is consistent with previous findings (35). As an important psychosocial resource, social support has consistently been associated with better psychological well-being and adaptation by providing emotional, informational, and practical assistance (36). Previous studies have further reported that greater social support is related to lower levels of psychological distress and more favorable mental health outcomes in populations with chronic conditions (35). These beneficial effects may be mediated through both psychological and neurobiological mechanisms, including modulation of the hypothalamic-pituitary-adrenal axis, noradrenergic activity, and oxytocinergic signaling (37). From an affective neuroscience perspective, resilience is characterized by the downregulation of negative affect, the upregulation of positive affect, and diminished self-referential processing (38). Social support likely exerts its influence across these domains by attenuating stress reactivity, enhancing positive emotional experiences, and reinforcing social connectedness. Therefore, the association observed in this study highlights the importance of considering social support when assessing psychological resilience among adults with prediabetes. Routine evaluation of patients’ family, peer, and community support resources may help identify individuals who are more likely to belong to lower resilience profiles and who could benefit from additional psychosocial support. Strengthening existing support networks and encouraging participation in family- or peer-based support programs may represent useful components of individualized nursing care.

In the present study, illness perception, as measured by the BIPQ, was not significantly associated with latent resilience profiles. This finding may partly reflect the relatively early stage of prediabetes. Unlike established diabetes, prediabetes is often asymptomatic and may not yet involve substantial treatment burden or disease-related complications, which may reduce the salience of illness perceptions (1). In addition, the brief format of the BIPQ may not fully capture the multidimensional and potentially subtle illness perceptions associated with prediabetes. It is also possible that illness perception influences psychological resilience indirectly through other psychosocial factors rather than exerting a direct association. These possibilities should be examined in future longitudinal studies.

The present findings further underscore the role of socioeconomic factors in shaping latent profiles of psychological resilience among individuals with prediabetes. Compared with self-paying participants, those covered by public medical insurance or other insurance schemes were significantly less likely to be classified into the low-resilience profile, suggesting that financial protection may represent as a critical resource associated with psychological resilience. Access to medical insurance may alleviate the economic burden associated with disease management, alleviates uncertainty regarding healthcare accessibility, and enhances individuals’ perceived security, which may in turn be associated with greater psychological resilience. In addition to financial resources, marital status was also associated with resilience profile membership. Compared with single individuals, divorced or widowed participants were less likely to be classified into the low-resilience profile. Although this finding may appear counterintuitive, resilience has been conceptualized as a dynamic process that may emerge through successful adaptation to major life adversities, including interpersonal loss and marital dissolution. Previous studies suggest that many individuals gradually regain psychological functioning following such experiences by developing more effective coping strategies and emotional regulation abilities (39) However, this association should be interpreted with caution, as divorced or widowed participants are generally older than single individuals, and age-related differences in life experience and emotional regulation may partly account for the observed association. In addition, within the Chinese cultural context, emotional and instrumental support from extended family members and broader social networks may remain available following marital dissolution, potentially contributing to psychological adaptation (40). Taken together, these findings highlight the need to address both individual and structural determinants of resilience in prediabetes management.

### 4.1 Limitations

This study has several limitations. First, the cross-sectional design limits causal inferences regarding the associations between latent profiles of psychological resilience and related factors among adults with prediabetes. Because all variables were assessed concurrently, the temporal direction and potential reciprocal relationships between psychological resilience and related psychosocial factors could not be determined and warrant further investigation using longitudinal designs. Second, reliance on self-reported measures may have introduced recall and social desirability bias, particularly among participants requiring interviewer-assisted completion. In addition, psychological resilience may not be fully captured by standardized scale-based assessments. Third, although several covariates were adjusted for, residual confounding from unmeasured factors, such as lifestyle behaviors, glycemic control, and mental health status, cannot be excluded. Fourth, as a data-driven method, LPA may yield profiles that are sensitive to sample characteristics and model specifications, potentially affecting their stability and reproducibility. Finally, the use of convenience sampling from a single tertiary hospital in Guangzhou and the relatively small sample size may have limited the representativeness and generalizability of the findings to the broader population of adults with prediabetes in China. Moreover, because characteristics of eligible individuals who declined participation were not systematically recorded, potential selection and non-response bias could not be directly assessed. Future multicenter studies with larger and more representative samples and longitudinal designs are warranted to validate and extend these findings.

### 4.2 Implications for future research

Future research should prioritize longitudinal and multicenter designs to examine the temporal stability, transition patterns, and potential reciprocal relationships between psychological resilience profiles and related factors among adults with prediabetes. Larger and more representative samples are also needed to validate the robustness and generalizability of the identified profiles and to reduce potential selection bias. In addition, incorporating objective clinical indicators, behavioral measures, and multi-source assessments may complement self-reported data and reduce measurement bias. Future studies should also consider a broader range of psychological, metabolic, and lifestyle factors to further elucidate the determinants and potential mechanisms underlying resilience heterogeneity. Finally, longitudinally validated profiles could provide a basis for developing and evaluating tailored, profile-specific interventions to promote psychological resilience and support long-term health management among adults with prediabetes.

## 5 Conclusions

This study identified three distinct latent profiles of psychological resilience among adults with prediabetes, revealing substantial heterogeneity within this population. Self-efficacy, social support, and socioeconomic-related factors, including medical payment method and marital status, were significantly associated with profile membership. These findings have important nursing implications for early identification of individuals with low psychological resilience in prediabetes care. Integrating psychological profiling into routine nursing assessment may facilitate risk stratification and support individualized care planning. Targeted nursing interventions aimed at enhancing self-efficacy and strengthening social support may improve psychological resilience and promote engagement in self-management behaviors, thereby contributing to long-term metabolic risk reduction. Overall, this study has provided evidence to support the development of stratified, profile-based nursing approaches for individuals with prediabetes.

## Data Availability

The datasets are not publicly available due to privacy and ethical restrictions but are available from the corresponding author on reasonable request.
